# Heat: A Highly Efficient Skin Enhancer for Transdermal Drug Delivery

**DOI:** 10.3389/fbioe.2018.00015

**Published:** 2018-02-15

**Authors:** Sabine Szunerits, Rabah Boukherroub

**Affiliations:** ^1^Univ. Lille, CNRS, Centrale Lille, ISEN, Univ. Valenciennes, UMR 8520, IEMN, Lille, France

**Keywords:** transdermal delivery, thermal activation, nanoparticles, skin, drug, patches

## Abstract

Advances in materials science and bionanotechnology have allowed the refinements of current drug delivery systems, expected to facilitate the development of personalized medicine. While dermatological topical pharmaceutical formulations such as foams, creams, lotions, gels, etc., have been proposed for decades, these systems target mainly skin-based diseases. To treat systemic medical conditions as well as localized problems such as joint or muscle concerns, transdermal delivery systems (TDDSs), which use the skin as the main route of drug delivery, are very appealing. Over the years, these systems have shown to offer important advantages over oral as well as intravenous drug delivery routes. Besides being non-invasive and painless, TDDSs are able to deliver drugs with a short-half-life time more easily and are well adapted to eliminate frequent administrations to maintain constant drug delivery. The possibility of self-administration of a predetermined drug dose at defined time intervals makes it also the most convenient personalized point-of-care approach. The transdermal market still remains limited to a narrow range of drugs. While small and lipophilic drugs have been successfully delivered using TDDSs, this approach fails to deliver therapeutic macromolecules due to size-limited transport across the *stratum corneum*, the outermost layer of the epidermis. The low permeability of the *stratum corneum* to water-soluble drugs as well as macromolecules poses important challenges to transdermal administration. To widen the scope of drugs for transdermal delivery, new procedures to enhance skin permeation to hydrophilic drugs and macromolecules are under development. Next to iontophoresis and microneedle-based concepts, thermal-based approaches have shown great promise to enhance transdermal drug delivery of different therapeutics. In this inaugural article for the section “Frontiers in Bioengineering and Biotechnology,” the advances in this field and the handful of examples of thermal technologies for local and systemic transdermal drug delivery will be discussed and put into perspective.

## Introduction

Topical remedies, such as creams, gels, ointments, and bandages, rubbed or applied to the skin, have been used over centuries. The well-known *Papyrus Ebers* (1550 C) is probably one of the oldest records containing the description of many drugs and formulations for the treatment of burns, wounds, blisters, and exudation. The concept that certain drugs can cross the skin might be traced back to Ibn Sina, a Persian physician, who proposed that dermally applied drugs can have a local effect, but can also affect tissue immediately beneath the skin as well as more remote areas, and can be considered as one of the most ancient consideration of transdermal drug delivery. Indeed, it was observed that next to the primary role of the skin serving as efficient barrier against the invasion of the organism by viruses, bacteria, dust, allergens, toxic chemicals, UV irradiation, and particulate materials (Roberts et al., 2002), some molecules can penetrate more deeply into the skin structure.

### Passive Delivery Modes

**Figure 1:**
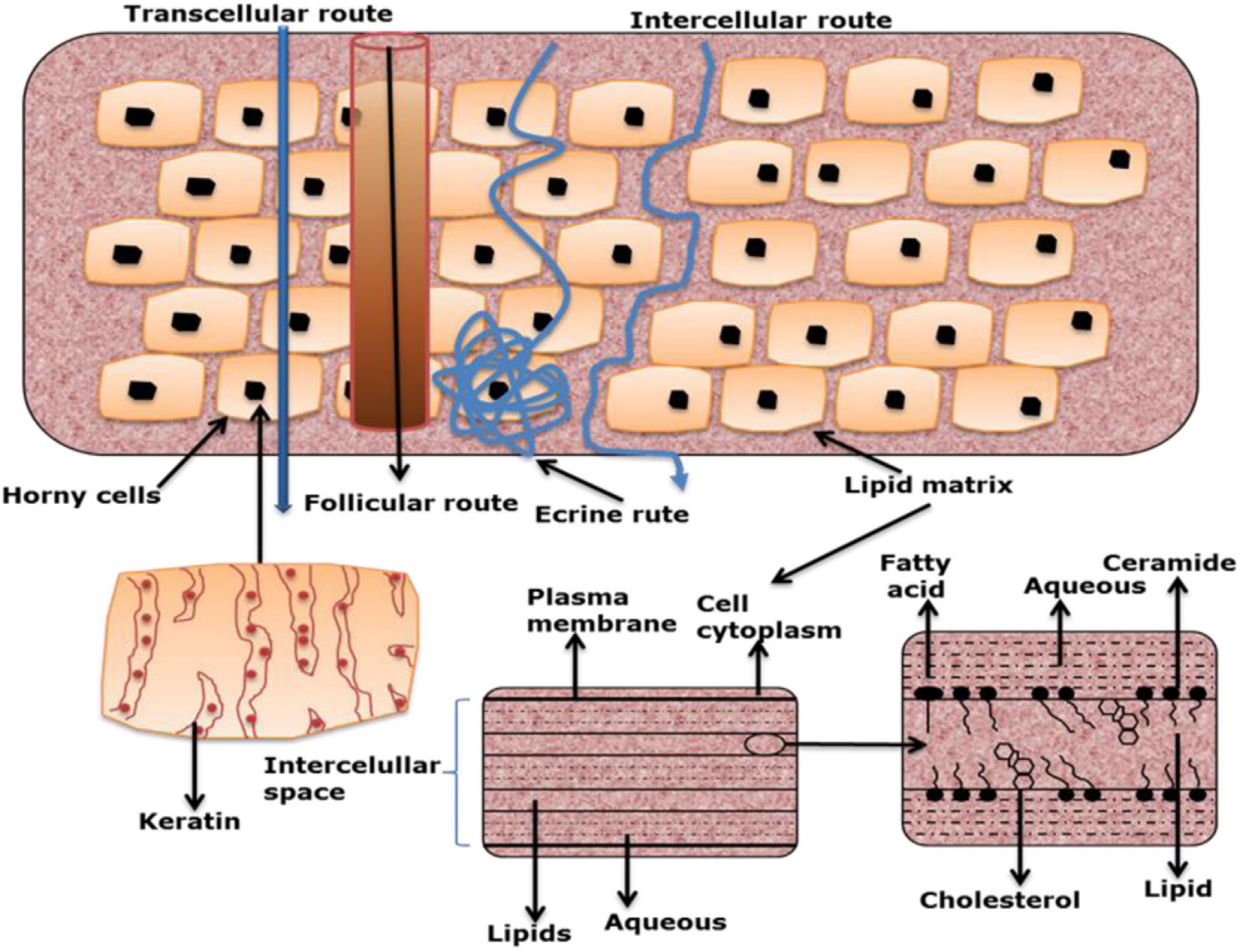
Transcellular and intercellular routes of drug delivery *via* the skin [reprint with permission from Escobar-Chávez et al. (2012)].

The skin is in fact relatively permeable to lipid-soluble substances, which can overcome the *stratum corneum* (SC) barrier, the outermost layer of the skin. Various cases of poisoning were reported in the early 1900s in France after topical applications of aniline dyes in dyed cloths and shoes. The first *in vivo* studies, at the beginning of the twentieth century, demonstrated undeniably the systemic absorption of certain molecules after topical application by analyzing the drug level in blood and urine. It has become clear that the hydrophobic lipids of the SC block the entry of most topically applied drugs, other than those that are lipid-soluble and of low molecular weight. For these small penetrants, the intracellular route is favored, where the therapeutics move freely within the inter-cellular space and diffusion rates are governed largely by the lipophilicity of the drug (Figure 1). It was also at this time that the first adhesive transdermal delivery systems were designed. Transdermal skin patches were seen to offer the potential to maintaining sustained steady-state blood levels after topical application, with the levels being carried by manipulating the drug concentration and vehicle components in the patch.

The next step was to identify transdermal drug candidates. From a physicochemical point of view, an ideal transdermal drug candidate has to meet a number of requirements (Table 1). Drugs such as nitroglycerin (treatment of angina pectoris), estradiol (female hormone replacement therapy), fentanyl (pain treatment), and scopolamine (treatment of motion sickness) have the necessary properties and are one of the first drugs which have been successfully developed into marketed transdermal systems. To this non-exhaustive list of drugs integrated into transdermal delivery patches has to be added the nicotine patch for smoking cessation aid, one of the first transdermal starts. Indeed, in only some months during the end of 1991, four nicotine patches with different designs reached the market. Over a million smokers gave up smoking with the help of these patches (Prausnitz et al., 2004) and helped transdermal patches for not only being widely accepted by the public, but actively demanded and researched for. Currently about 20 drugs have been FDA-approved for transdermal administration sharing several characteristics such as low molecular weight, lipophilicity, and relatively low-dose administration requirements. Several other drugs have been tested but never made it to the market. Indeed, despite encouraging results on using transdermal timolol ointments to treat increased pressure inside the eye or captopril incorporated patches, an angiotensin-converting enzyme inhibitor, none of these patches has received clinical and consequently regulatory acceptance. In the case of captopril, the physicochemical properties were not favorable for transdermal delivery as the drug was associated with severe skin irritation (Helal and Lane, 2014). The other limitation of such passive transdermal patches was encountered with macromolecular therapeutics such as insulin and other proteins. Indeed, none of these therapeutics has been made it to a marketable transdermal patch yet.

**Table 1:** Required physicochemical properties for a passive transdermal drug delivery and FDA-approved transdermal delivery systems.

Physicochemical properties	Requirements for passive transdermal drug delivery		
Liphophilic character	High lipophilicity		
Melting point	<250°C		
Molecular weight	<500 Da		
Log P (octanol/water partition coefficient)	1–5		
Toxicity	No local skin reactions		
Water solubility	≈0.05 to 1 mg/mL if target delivery rate is in the mg/day range		

**Drug**	**Molecular weight/Da**	**Indication**	**Year**

Buprenorphine	467	Chronic pain	2010
Capsaicin	305	Neutropathy pain	2009
Clonidine	230	Hypertension	1984
Diclofenac epolamine	411	Acute pain	2007
Estradiol	272	Menopausal symptoms	1986
Estradiol/levonorgestrel	272/312	Menopausal symptoms	2003
Estradiol/norethidrone	272/341	Menopausal symptoms	1998
Ethinyl estradiol/norelgestromin	296/327	Contraception	2001
Fentanyl	337	Chronic pain	1990
Granisetron	312	Chemo-induced emesis	2008
Influenza-virus vaccine	122,000	Influenza virus	2011
Lidocaine with tetracaine	234/264	Local dermal analgesia	2004
Methylphenidate	233	Hyperactivity disorder	2006
Nicotine	162	Smoking cessation	1991
Nitroglycerin	227	Angina pectoris	1981
Oxybutynin	358	Overactive bladder	2003
Rivastigmine	250	Dementia	2007
Rotigotine	316	Parkinson’s disease	2007
Scopolamine	303	Motion thickness	1979
Selegiline	187	Depressive disorder	2006
Testosterone	288	Testosterone deficiency	1993

To increase the list of therapeutics that can be delivered through the skin, significant efforts have been devoted to the development of new approaches to enhance transdermal delivery. The use of chemical skin enhancers (e.g., propylene glycols, oleic acid, azone, *N*-methyl pyrrolidone, Tween 80, limonene, etc.) (Finnin and Morgan, 1999; Pathan and Setty, 2009; Williams and Barry, 2012) has allowed, in several cases, to increase passive diffusion of small therapeutics, but has not been useful in overcoming the size-limiting factor of drugs (Chen et al., 2006; Kim et al., 2007; Som et al., 2010; Zakrewsky et al., 2014). A further common drawback of permeation enhancers is that their efficiency is often closely mimicked by skin irritation due to their mechanism of action, disrupting the ordered SC lipid bilayers or corneocytes structure organization (Thong et al., 2007; Williams and Barry, 2012; Herman and Herman, 2015).

Considerable efforts have been made on the transdermal delivery of therapeutics using nanoparticles (NPs), including lipid-based NPs, liposomes, niosomes, transfersomes, ethosomes, dendrimers, micellar NPs, polymeric as well as metallic and magnetic nanostructures (Jijie et al., 2017). The exact mechanism by which NPs improve transdermal delivery of drugs is still not fully clarified, but it is assumed that NPs increase the permeation of the drugs by increasing their aqueous solubility through disruption of the well-organized structure of skin by interacting with skin lipids and/or protein structures. Small NPs, in particular smaller than 50 nm in diameter, can in general more easily penetrate the skin, resulting in better permeation profiles (Kohli and Alpar, 2004).

### Enhanced Delivery Modes

**Figure 2:**
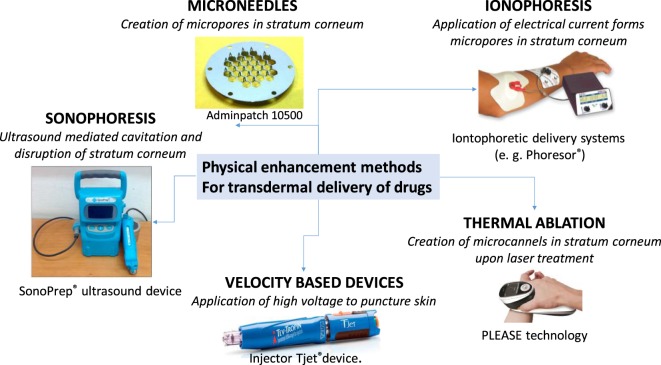
Physical enhancement approaches for transdermal drug delivery together with some examples of commercially available products and their mode of action.

The other concepts widely used are based on the application of external physical triggers to actively increase skin permeabilization (Figure 2). The application of adhesive tapes or cyanoacrylate glue, known as stripping, is probably the oldest physical method to allow drug delivery. This approach removes both corneocytes and extracellular lipids, reducing the path length that drugs need to cross.

Pressure-driven jets have been investigated for intradermal delivery of a variety of drugs and enabled delivering of controlled doses of medication (Schramm and Mitragotri, 2002). The threshold velocity for jet penetration into human skin was around 100–200 m/s to puncture skin. This concept is currently used for the delivery of somatropin, a human growth hormone and commercialized under the name Tjet^®^. Despite their introduction into clinical medicine, variability and occasional bruising have limited their widespread acceptance. The risk of cross-contamination is, however, highly elevated and patients have reported pain (Jackson et al., 2001).

Iontophoretic skin patches, using physiologically acceptable electrical currents (0.1–1 mA cm^−2^) applied for minutes to hours from an externally placed electrode in order to drive the drugs across the SC, have shown some success for drugs that can benefit from the use of an electric current to increase the speed or rate of delivery such as lidoacaine/epinephrine, fentany, or most lately a drug against migraine, sumatriptane, known under the commercial name of Zecuity. The transdermal transport rate is proportional to the applied constant current, enabling enhancement of transdermal dose and control of drug delivery kinetics. The amount of drug delivered is determined by the maximal current applicable before the pain level is reached. This approach is, however, not adapted to the delivery of larger molecules. Pores can be also formed through the application of short and high voltages (50–500 V), commonly known under electroporation. Properly designed systems can minimize sensation from the pulses and facilitate drug delivery, especially of hydrophilic and charged molecules into the skin. While small and higher molecular weight drugs can be delivered into the skin, the main drawbacks are the lack of quantitative delivery, cell death with damage of proteins and thus their bioactivity. This approach is only at the research stage with regard to transdermal delivery.

The use of low-frequency ultrasound for the transdermal delivery of lidocaine, a local anesthesia, was approved in 2004 (Som et al., 2010). Cavitational ultrasound, using frequencies <1 MHz, is based on the generation of microbubbles, which can mechanically impact the skin by creating submicroscopic defects in SC. This concept has been approved as a pretreatment prior to the application of lidocaine as a means of accelerating local anesthesia. However, high-intensity ultrasound causes second-degree burns, limiting the delivery of macromolecules (Szinger et al., 1998). Ultrasounds have been widely applied as transdermal delivery systems for the treatment of many diseases and lately also proposed in combination with microneedles (MNs) for enhanced drug permeation (Han and Das, 2015).

Microneedle arrays have shown to offer a highly promising solution for overcoming the skin barrier and to deliver small molecular as well as macromolecular therapeutics such as proteins, peptides, and vaccines (Larrañeta et al., 2016a; Ripolin et al., 2017). Indeed, one of the main advantages of MNs is that a wide variety of drugs can be successfully delivered transdermally and intradermally (Prausnitz, 2004; Donnelly et al., 2012; Tuan-Mahmood et al., 2013; Larrañeta et al., 2016b). Since the first proof-of-concept of such a device for enhanced drug delivery into the skin (Henry et al., 1998), a large growing body of literature has investigated various microfabrication technologies for MN array fabrication, including numerous materials next to silicon such as metals and various polymers and more lately hydrogels. Two MN arrays are on the market and known under the name AdminPatch^®^ 1500 and 1200. These MN arrays consist of 31 MNs of 1,200–1,500 µm in length located within 1 cm^2^ circular area. The entire device is 20 mm in diameter and is made of medical-grade SS316L stainless steel. Zosano Pharma Corporation has developed a transdermal MN product based on solid titanium MNs coated with recombinant human parathyroid hormone 1-34, teriparatide (PTH, 4.1 kDa), for the management of osteoporosis (Daddona et al., 2011). The titanium MN arrays were produced by photochemical etching. This patch was discontinued in 2015; however, the company recently announced that outcomes from a phase-III trial into the delivery of zolmitriptan met both primary endpoints. A MN-based vaccine is available from Nanopatch™, Vaxxas Pty Ltd.

While MNs have shown strongly enhanced drug delivery, the use of silicon or metal MNs has obvious issues with biocompatibility and broken silicon or metal MN can cause skin damage. Furthermore, coating solid MN is not an easy task and these coatings only deliver a very small drug amount. While some of these limitations can be overcome using polymeric MNs (chitosan, cellulose, hyaluronic), most of the polymers dissolve rather quickly upon contact with the skin interstitial fluid and drug is released fast. Sustained release of BSA for at least 68 days was demonstrated by Chen using dissolvable chitosan-based MN patches (Chen et al., 2012). This approach is indeed adapted for the delivery of macromolecules and a dissolving MN array based on hyaluronic acid loaded with insulin has been proposed by Yamamoto and coworkers (Liu et al., 2012). The length of the MNs was 800 µm with a base diameter of 160 µm and a tip diameter of 40 µm, maintaining the skin piercing ability for 1 h even et elevated humidity of 75%. Commercial dissolvable MNs are currently developed by Corium Inc. [MicroCor^®^ PTH (1–34)] and BioSerenTach Inc. (lixisenatide), but are still at an early stage of development.

Hydrogel-forming MN arrays might be an alternative and are currently under investigation. The group of Donnelly described the first hydrogel-forming MN array in 2012 using aqueous blends of polymeric materials, which are poured into silicon micro-molds filled using laser drilled silicon (Donnelly et al., 2012). Curing the gel at 80°C for 24 h and removing the gel from the mold resulted in hydrogel MN arrays. Drug-loaded patches were attached to the base of the MN array to form an integrated transdermal delivery system. Upon application onto the skin, water diffuses into the MN array, resulting in swelling of the hydrogel and liberation of the drugs without destruction of the array. Different drugs including macromolecules such as BSA and insulin could be permeated using this approach (Donnelly et al., 2012). Further studies by the same group demonstrated that transdermal drug delivery can be easily controlled by modulating the cross-link density of the hydrogel matrix. This indicates that drug delivery can be tailored on a case-by-case basis to meet the requirements of different drugs with different therapeutic windows (Garland et al., 2012). Super swelling hydrogel MNs were proposed lately by the same group as an alternative MN system with the advantage of the needles remaining intact even after skin permeation. This system was made from an aqueous blend containing PEG10.000/Na_2_CO_3_ and Grantrez S-97 and delivered 44 mg of ibuprofen sodium in 24 h (Donnelly et al., 2014). Microwave-assisted cross-linking of PEG and poly(methyl vinyl ether-alt-maleic acid) was lately proposed by the same group as an alternative to thermal cross-linking, being several times faster, but showing the same release profiles for caffeine (Larraneta et al., 2015).

In addition to these different approaches, heat-assisted microporation to create transport channels in the skin to enable controlled enhanced drug delivery has become a well-accepted approach and considered ideally adapted for the delivery of macromolecular therapeutics. The availability of different medical lasers was very fruitful for the development of transdermal delivery systems as the applied laser energy per unit area (fluence) controls the death of each formed micropore with the possibility of some special configurations to form hundreds of micropores with controlled depth in a few seconds. Since the number of pores created in the skin and the depth of the pores can be easily varied, this approach provides a simple means to control the drug dose delivered through the skin and to develop personalized therapy. Other microporation technologies lack indeed this flexibility. A closer look will thus be given to laser-based skin ablation for transdermal drug delivery in the following.

## Heat-Based Ablation of Skin for Transdermal Drug Delivery

### Direct Laser Ablation Enhancement

Ablation of the SC by mechanical abrasion, tape-stripping, or chemical treatment is known to enhance drug permeation. However, this approach is limited due to a lack of control and reproducibility and the potential to cause pain and skin irritation. Thermal ablation, based on the selective removal of the SC by localized microsecond heat pulses, is a promising alternative to increase the permeability of the skin’s outer barrier layer while sparing deeper living tissue (Levin et al., 2005; Lee et al., 2011a). The creation of local heat leads to cell ablation and transient creation of microchannels or pores typically 50–100 µm in diameter. This technology enables the transdermal delivery of a wide range of drugs including macromolecules. Next to radiofrequency (Sintov et al., 2003) and ultrasound-based heating (Azagury et al., 2014), much attention has been given to the use of lasers for thermal skin ablation as a precise control on the ablated skin depth can be achieved upon tuning the pulse duration and the wavelength of the laser used. In the case of ultrasound-based approaches, the proposed mechanism by which ultrasound affects tissues and cells include, next to cavitation effects, thermal effects (Azagury et al., 2014). Ultrasound can increase the temperature of the skin by the absorption of the sound wave with a frequency greater than the upper limit of human hearing range. The higher the medium’s absorption coefficient, the higher the temperature increase and the generated thermal effect. In the case of radiofrequency-based ablation, a thin needle like electrode is placed directly into the skin. Application of high-frequency alternating current (100 kHz) results in the formation of microchannels, while exposure to higher frequencies (100–500 kHz) causes ionic vibration within the tissue and local heating of the skin with ablation of the cells in that region, resulting in enhanced drug transport across the skin (Sintov et al., 2003; Arora et al., 2008).

When it comes to laser-based thermal ablation, many laser types with a broad wavelength range have been tested (Table 2), but only a few can be applied to transdermal delivery, for which little tissue damage due to thermal effects is required (Lee et al., 2001, 2002, 2011b; Fang et al., 2004; Gomez et al., 2008; Bachhav et al., 2010, 2011; Li et al., 2013).

**Table 2:** Lasers used for transdermal drug delivery *via* skin ablation.

Laser	Wavelength/nm	Pulse duration	Role in transdermal delivery
CO_2_ laser	10,600	50 ms	Ablation *via* vaporization
Er:YAG	2,940	250–400 µs (traditional)10–300 µs (fractional)	Dermal removalFractional epidermal removal
Laser-diode laser	808 and 980	1 ms–15 min (continuous)	Photothermal ablation
Nd:YAG	650–1,100	15 ns	Photothermal ablation
Ti:sapphire	650–1,100	15 ns	Photothermal ablation

**Figure 3:**
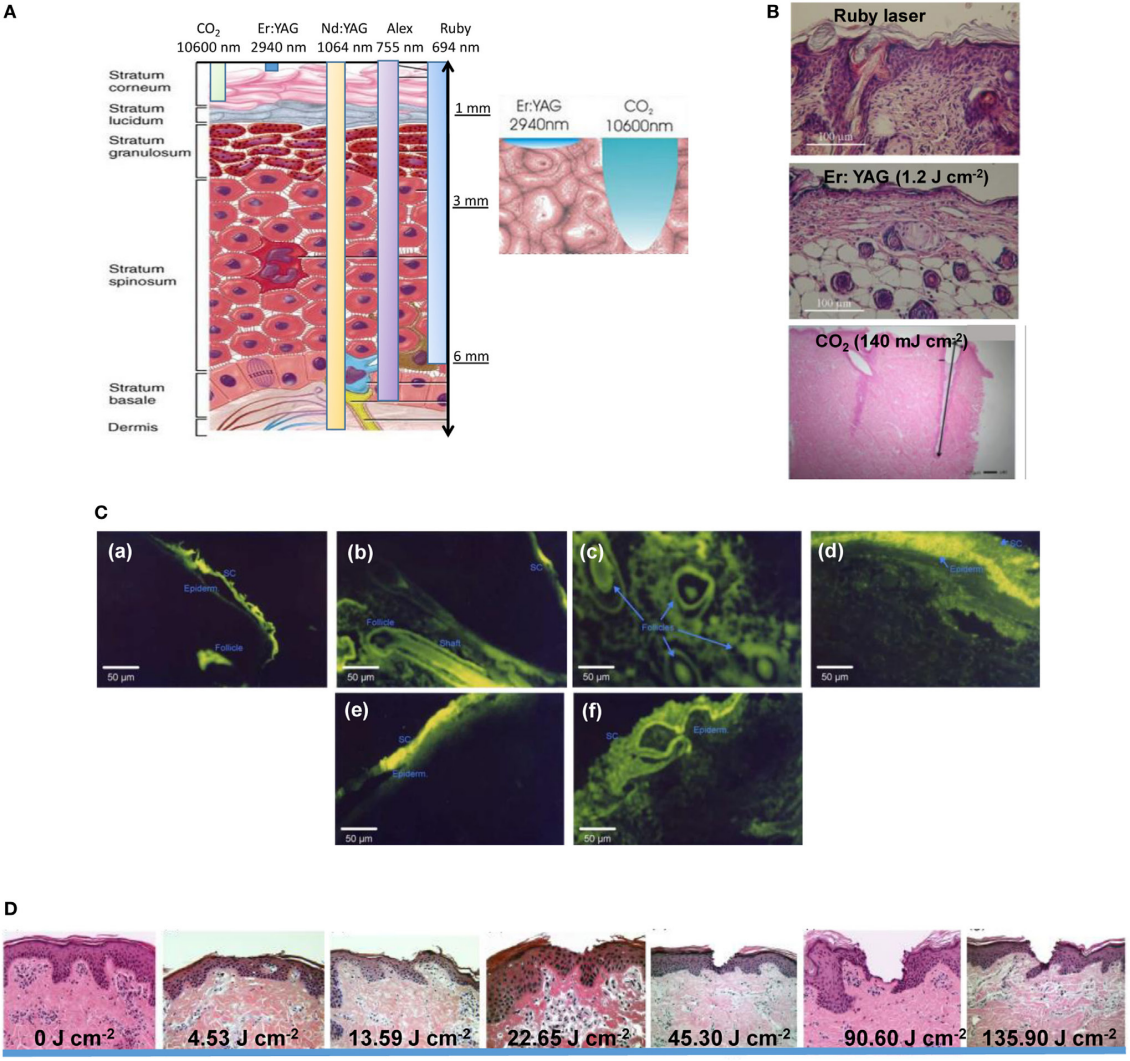
**(A)** Penetration depth through the skin tissue of different lasers [accessed at http://www.dekalaser.com/img/ENGskinsurgery_02.jpg (15 December 2017)]. **(B)** Histological examination of nude mouse back skin treated with ruby laser, erbium–yttrium–aluminum–garnet (Er:YAG) (1.2 J cm^−2^) and CO_2_ (140 mJ cm^−2^) lasers. **(C)** Fluorescence microscopy examination after topical administration of FITC and FITC-labeled dextrans *via* pig skin for 30 min: (a) topical FITC delivery into the skin treated by Er:YAG laser at 1.7 J cm^−2^ in the longitudinal section, (b) FITC delivery into the skin treated by Er:YAG laser at 1.7 J cm^−2^ in the cross-section, (c) FITC-dextran (4.4 kDa) delivery passively, (d) FITC-dextran (4.4 kDa) delivery into the skin treated by Er:YAG laser at 1.7 J cm^−2^ in the longitudinal section, (e) FITC-dextran (38 kDa) passive delivery, and (f) FITC-dextran (38 kDa) delivery into the skin treated by Er:YAG laser at 1.7 J cm^−2^ in the longitudinal section. **(D)** Hematoxylin/erosin-stained histological sections of porcine skin samples: (a) untreated and after painless laser epidermal system P.L.E.A.S.E. treatment at different fluences (J cm^−2^): (b) 4.53, (c) 13.59, (d) 22.65, 45.3, (e) 90.6, and (f) 135.9 together with cumulative lidocaine permeation across P.L.E.A.S.E. porated skin [reprint with permission from Bachhav et al. (2010)].

The ruby laser and the alexandrite laser, belonging to the near-infrared (NIR) lasers (650–900 nm), are commonly used to remove pigmentation of hair, but are not sufficient to remove the SC (Figure 3A) and are not used for transdermal delivery. Indeed, visible red light is readily absorbed by blood and skin surface components, thereby limiting its tissue penetration to <10 mm. The light of the NIR titanium: sapphire laser is not readily absorbed by the skin and has a much larger depth of tissue penetration of 30–40 mm (Gupta et al., 2014). However, the heating effects are low and no skin ablation might occur. By contrast, the wavelengths of the erbium–yttrium–aluminum–garnet (Er:YAG) as well as the CO_2_ laser, located above 1,400 nm in the far-infrared, induce heating and microporation of the skin through water excitation and explosive evaporation of the epidermis (Figure 3B). The CO_2_ laser results in very sharp and deeply penetrated tissue ablation with narrow peripheral thermal necrosis. The output of an Er:YAG laser is strongly absorbed by water, which results in about 15 times better light absorption by the skin and favored properties for enhanced transdermal drug delivery when compared with the CO_2_ laser. A comparison of ruby, CO_2_, and Er:YAG laser on the skin permeability of 5-fluorouracil showed that while the ruby laser only moderately enhanced the drug flux (Lee et al., 2002), the Er:YAG laser with a fluence of 0.8–1.4 J cm^−2^ enhanced the flux of 5-fluorouracil by 53–133 times than untreated skin, while the CO_2_ laser increased the 5-fluorouracil penetration by 36–41 times only under the fluence of 4–7 J cm^−2^. This suggests that Er:YAG lasers at low fluence can safely and painlessly enhance drug absorption by the skin (Lee et al., 2001, 2008a; Fang et al., 2004). Using fluorescein isothiocyanate-labeled dextrans of increasing molecular weight (4.4, 49.4, 38, and 110 kDa) in combination with fluorescence microscopy allowed, in addition, the examination of the distribution of the drug in the different skin layers, depending on the treatment (Figure 3C) (Fang et al., 2004). The results indicate a significant increase in the permeation of dextran across the skin after laser treatment. Most importantly, while ablation of the SC layer, photomechanical stress on intercellular regions and alterations of the morphology and arrangements of corneocytes were proposed as a possible mechanism how the Er:YAG laser promotes macromolecular drug delivery; no alternation of viable skin morphology was observed after laser treatment and the partial ablation of the SC was found to be reversible. Even insulin with a MW of 38 kDa passed the skin barriers upon laser treatment.

For thermal ablation-enhanced transdermal drug delivery, higher laser energies with pulse durations less than microsecond are ideal to limit heat transfer to surrounding tissue. Indeed, while the skin surface is extremely hot, the viable epidermis and deeper skin tissue is not, largely eliminating side effects and fast improved safety. A laser microporation technology called P.L.E.A.S.E. (Precise Laser Epidermal System; Pantec Biosolutions AG, Lichetenstin) has been proposed by Kalia and coworkers for enhanced drug delivery through the skin (Bachhav et al., 2010, 2011; Yu et al., 2011). The P.L.E.A.S.E. device is built around a diode-pumped fractional Er:YAG laser. In contrast to conventional Er:YAG lasers that ablate a 7-mm spot on the skin, the device uses a specially designed scanning laser to create a user-defined array of micropores in the skin surface where the depth of each micropore is controlled by the fluence. In principle, increasing the number of micropores increases the number of transport channels and should therefore increase drug delivery rates. In addition, the pulse duration is shorter to ensure the localization of the heat transfer to the skin surface without heat propagation. Histological sections confirmed that low-energy application (4.53–13.59 J cm^−2^) resulted in selective removal of the SC (20–30 µm), intermediate energies (22.65 J cm^−2^) produced pores that penetrated the viable epidermis (60–100 µm), while higher energies created pores that reached the dermis (>150–200 μm) (Figure 3D).

Table 3 gives an overview of the different drugs delivered using laser-based thermal ablation. Indeed, one of the main advantages over other technologies is that the approach is adapted for hydrophilic macromolecular compounds such as dextran, as well as antibodies such as anti-thymocyte globulin (ATG) used to the induction of immunosuppression, but also for the delivery of DNA and RNA molecules. Next to MNs, it remains the only approach for transcutaneous protein delivery. Application of OVA using P.L.E.A.S.E. technology induced equal to or higher immune responses compared with immunization by subcutaneous injection.

**Table 3:** Drugs transdermally delivered using laser ablation technology.

Small drugs	Molecular weight/Da	Laser source	Reference
5-Aminolevulinic acid	131	Fractional Er:YAG	Shen et al. (2006)
Diclofenac	296	Er:YAG	Bachhav et al. (2011)
5-Fluorouracil	130	Er:YAG	Lee et al. (2002) and Gomez et al. (2008)
Lidocaine	234	Fractional Er:YAG	Bachhav et al. (2010)
Methotrexate	454	Er:YAG	Lee et al. (2008a)
Vitamin C	176	Er:YAG	Lee et al. (2003)

**Macromolecular drugs**	**Molecular weight/kDa**		

Anti-thymocyte globulin	37	Fractional Er:YAG	Yu et al. (2011)
β-Galactosidase	50	Fractional Er:YAG	Weiss et al. (2012)
Bovine serum albumin	70	Fractional Er:YAG	Lee et al. (2008b)
Dextran	4–150	Fractional Er:YAG	Fang et al. (2004)
Insulin	38	l Er:YAG	Fang et al. (2004)
RNA	9	Er:YAG	Lee et al. (2009)

A combination of laser ablation of SC with electroporation, producing transient pores within the lipid bilayers of the SC, was more recently reported to increase the delivery of methotrexate (MTX), a highly hydrophilic and high molecular weight (MW = 454.56 Da) agent used for the treatment of human skin diseases such as psoriasis or an immune suppressant in the treatment of rheumatoid arthritis *via* the skin. A combination of laser pretreatment and electroporation results in a 5- to 6.6-fold higher flux of MTX using a combination of 1.4 J cm^−2^ and 10 electroporation pulses over transport by laser or electroporation only.

### Photothermal Enhancement Strategy for Drug Delivery Using Nanomaterials

**Figure 4:**
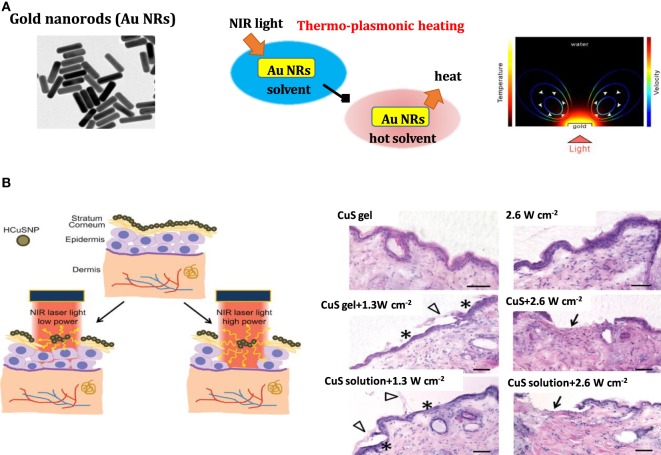
**(A)** Plasmonic heating: (left) TEM image of gold nanorods (Au NRs) (middle) effect of thermo-plasmonic heating on the temperature of the surrounding medium and (right) temperature map on Au NRs upon light illumination [reprint with permission from Baffou et al. (2013)]. **(B)** (left) Illustration of near-infrared (NIR) laser-triggered photothermal ablation of skin mediated by hollow copper sulfide (CuS) nanoparticles (NPs) and (right) histological images of skin section of nude mice treated with hollow CuS NP gel, pulsed laser (2.6 W cm^−2^), hollow CuS NP gel + laser pulse (1.3 W cm^−2^), hollow CuS NP gel + laser pulse (2.6 W cm^−2^), hollow CuS NP solution + laser pulse (1.3 W cm^−2^), and hollow CuS NP solution + laser pulse (2.6 W cm^−2^); asterisks: epidermis without *stratum corneum*; arrowheads: *stratum corneum* layers stripped from epidermis; arrows: dermis with removal of both *stratum corneum* and viable epidermis; scale bars: 50 µm [reprint with permission from Ramadan et al. (2012)].

The development and advances made in the synthesis of nanomaterials were also beneficial for recent advancements in transdermal drug delivery approaches. In particular, the engineering of NPs for photothermal therapy (PTT) has become of high interest. Gold nanostructures such as nanorods (Au NRs) with maximal absorption in the NIR are of particular interest as photothermal agents (Figure 4) (Norman et al., 2008; Akhavan and Ghaderi, 2013; Kim and Kim, 2014; Mackey et al., 2014). Upon illumination of the particles with a wavelength comparable to or smaller than the particle size, surface plasmon confinement in the form of localized surface plasmon resonance (LSPR) occurs. The position of the LSPR wavelength is greatly dependent on the diameter, shape of the particles, and the refractive index of the surrounding medium. In the case of gold nanospheres, the plasmon resonance frequency can be tuned within the visible spectrum by changing the diameter from 30 to 300 nm. For anisotropic nanostructures such as Au NRs, nanocages, and nanocubes, the plasmon resonance can be tuned from 600 to 1,400 nm by varying the aspect ratio. Au NRs display two absorption peaks: one correlated with the short transversal axis at around 520 nm and the other one with the longer longitudinal axis situated between 640 and 1,000 nm, depending on the aspect ratio (length/width) of the nanostructures (Figure 4A). Localized surface plasmons have two effects: electrical field enhancement near the particles’ surface and the occurrence of an absorption maximum at the plasmon resonant frequency. One of the consequences of the strong electromagnetic field enhancement around plasmonic nanostructures is local heat generation. Laser pulses absorbed by the plasmonic nanostructures excite free electrons in the plasmon band, creating a pulse of hot electrons. The rapid relaxation of these exited electrons through electron–phonon interactions produces strong localized heat within ≈1 ps. Heat is then transferred from the NP to its surrounding through phonon–phonon interactions at a time scale of ~100 ps, and the nanosystem returns to its initial state (Figure 4A). When a continuous wave laser source is used, heat generated in the lattice of the gold nanostructures is continuously dissipated into the surrounding environment, resulting in an increase in temperature of the adjacent medium by tens of degrees. While the first demonstrations of plasmonic-heating at the beginning of the twentieth century were related to visible and later to NIR light PTT, more recently a surfactant:protein/Au NRs complex gas has been applied for transdermal delivery of proteins (Pissuwan et al., 2011).

Kim et al. (2015a) proposed soft block copolymer micelles containing a phase change material (lauric acid) and Au NPs in the core as a promising carrier for non-invasive transdermal delivery of drugs. The photothermal effect of the Au NPs encapsulated in block copolymer under visible light irradiation (520–530 nm, 30 mW cm^−2^) together with the integration of a temperature sensitive material such as lauric acid proved to be effective for controlled release of indomethacin from these nanostructures through the skin. Moreover, the *in vitro* studies using Franz diffusion cells and *in vivo* experiments on Albino guinea pigs proved that the hybrid systems ensure a deeper penetration of the indomethacin after 10 min of irradiation treatment without inducing thermal damage of the skin.

Semiconductor copper sulfide (CuS) NPs were proposed by Ramadan et al. for transdermal delivery (Figure 4B) (Ramadan et al., 2012). Irradiation with a nanosecond-pulsed Nd:YAG laser in tandem with a Ti:sapphire laser (900 nm) was used to induce rapid heating of the NPs with instantaneous heat conduction. The average temperature of the irradiated skin is only increased to 40–50°C. The depth of skin perforation can be precisely controlled by adjusting the laser power and helped in increasing the permeability of human growth hormone, offering an appealing opportunity for macromolecular drug and vaccine delivery.

**Figure 5:**
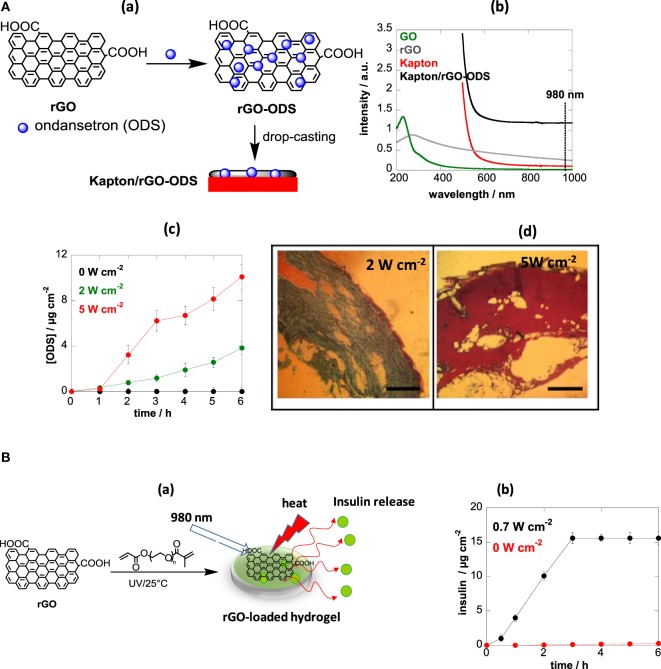
**(A)** (a) Fabrication of a drug-loaded reduced graphene oxide (rGO)-modified skin patch, (b) UV/Vis spectra of graphene oxide (GO), rGO, Kapton and Kapton/rGO-ondansetron (ODS), (c) *in vitro* permeation profiles of ODS through porcine skin from Kapton/rGO-ODS patches formed by mixing 500-µg/mL ODS with 1-mg/mL rGO upon light irradiation for 10 min using a continuous wave laser at 980 nm at different laser power densities, and (d) histology of pig ear skin after treatment with different laser power densities at 980 nm for 10 min. Scale bar = 0.5 mm [reprint with permission from Teodorescu et al. (2017) and Teodurescu et al. (2017)]. **(B)** (a) Formation of insulin-loaded photothermal active hydrogel and (b) *in vitro* permeation profiles of insulin [reprint with permission from Teodurescu et al. (2017)].

An alternative photothermal material recently proposed is reduced graphene oxide (rGO) (Yang et al., 2013). The strong optical absorption across the NIR spectrum of water-soluble polyethylene glycol modified rGO coupled with high chemical and thermal stability allowed rapid temperature rise and efficient way of heating (Yang et al., 2013). While rGO-based nanocomposites have been mainly proposed for cancer theranostics (Yang et al., 2012; Lim et al., 2013; Shi et al., 2013; Xu et al., 2013), as well as for the ablation of pathogens (Wu et al., 2013; Turcheniuk et al., 2015), we have demonstrated recently their interest for photothermal-based transdermal delivery of small and macromolecular compounds (Figure 5) (Teodorescu et al., 2017; Teodurescu et al., 2017). One approach consisted in impregnating rGO nanosheets with ondansetron (ODS) and depositing the matrix onto a flexible polyimide-based (Kapton) interface (Figure 5Aa). The loading mechanism of ODS onto rGO is believed to occur through π−π stacking and/or charge interactions between rGO and the positively charged pyridine network of ODS, although other contributions such hydrogen bonding and/or van der Waals interactions cannot be excluded. The formed Kapton/rGO-ODS interface exhibited strong light absorption at 980 nm (Figure 5Ab). Using a medical continuous laser at 980 nm, the affinity of ODS to the rGO can be altered, due to heat generated on the interface, and subsequently released. *In vitro* release profiles of ODS from the Kapton/rGO-ODS patches deposited onto pig skin indicated a correlation between the laser power density and the quantity of ODS crossing the skin. The ODS flux across pig skin at 5-W cm^−2^ irradiation was determined to be *J* = 3.1 µg cm^−2^ h^−1^ for the first 3 h and decreased to *J* = 1.6 µg cm^−2^ h^−1^ thereafter (Figure 5Ac). No significant histological changes were observed up to a laser power density of 2 W cm^−2^. In the case of 5 W cm^−2^, disruption of the SC was observed, which is in line with the enhanced transdermal ODS flux (Figure 5Ad). Addition of penetration enhancers such as Tween 20 into the patch significantly enhanced the ODS flux to 13.2 ± 1.5 µg cm^−2^ h^−1^. With a skin patch of 25 cm^2^, about 2 ± 0.2 mg of ODS are delivered every 6 h, which represents a therapeutically correct dose. This example shows thus, for the first time, the double action of light: heat generation to change the affinity of the drug to the patch with subsequent enhanced transdermal delivery.

This approach was proven to be also valid for the delivery of larger therapeutics such as insulin from hydrogels (Figure 5B) (Teodurescu et al., 2017). The 3D structure of the hydrogel showed to be favorable for the integration of insulin due to interaction with the porous hydrophilic network of the PEG-based hydrogel network. The temperatures reached in the patches upon light illumination were high enough to interfere with the affinity between human insulin and rGO, resulting in its release. Using such insulin-loaded photothermal active hydrogels (Figure 5Ba), it was demonstrated that the affinity of insulin inside the hydrogel can by modulated upon NIR irradiation with a permeation of insulin taking place at a flux of *J* = 5.8 ± 0.2 µg cm^−2^ h^−1^ in a relatively short time scale (0–3 h), significantly higher than passive diffusion (Figure 5Bb).

### Light-Responsive Microneedles

**Figure 6:**
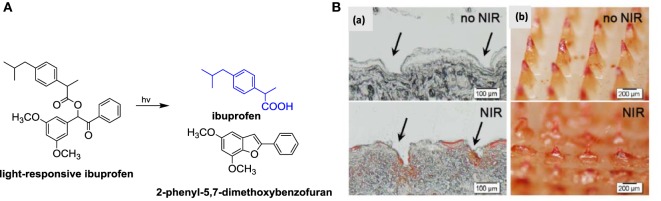
**(A)** Synthesis of light-responsive ibuprofen conjugates together with light-triggered release of ibuprofen from synthetic light-responsive conjugate. **(B)** (a) Histological sections and (b) bright-field micrographs of microneedles being removed from the inserted skin. The arrows indicate the microneedle insertion sites [reprint with permission from Chen et al. (2015)].

As discussed above, the minimally invasive nature of MNs together with the possibility to load small as well as macromolecular drugs have made them intensively investigated transdermal delivery platforms. However, they are currently mainly designed to deliver of bolus doses in relatively short times. On-demand delivery from MN systems has only recently been achieved using light-responsive hydrogels. Indeed, Hardy et al. developed MNs by micromolding 2-hydroxyethyl methacrylate (HEMA) and ethylene glycol dimethacrylate (EGDMA) in the presence of 3,5-dimethoxybenzoic acid as light-responsive conjugate (Figure 6A) (Chen et al., 2015). The obtained MN displayed excellent mechanical properties and can be loaded with up to 5% w/W of ibuprofen. *In vitro*, the system was able to deliver up to three doses of 50 mg of ibuprofen upon application of an optical trigger, a 15 W Hg discharge UV lamp source at 365 nm over a prolonged time period of up to 160 h.

Visible light-triggered on-demand drug release from hybrid hydrogel beads was reported by Kim et al. (2015b). Irradiation with visible light resulted in light-induced volume change of the temperature-responsive magnetic poly-*N*-isopropylacyrylamide-co-vinyl-2-pyrrolidone gel and release of dexamethasone (Hardy et al., 2016). The use of NIR-responsive composite MNs was also demonstrated by Chen et al. (2015). Silica-coated lanthanum hexaboride nanostructures were incorporated into MNs serving as an NIR absorber (Figure 6B). Light-to-heat transduction using an 808-nm continuous laser results in melting of the MNs at 50°C, increasing the mobility of the polymer chains and enabling drug release from the matrix. The *in vivo* applicability of the NIR-responsive MNs to anticancer drug delivery was demonstrated for DOX-loaded MNs applied to rat skin and irradiated for four laser on/off cycles.

## Conclusion and Perspectives

Transdermal delivery systems have become a successful alternative for a continuous drug delivery on demand. Transdermal patches can be removed in the event of adverse drug reactions or if the therapy has to be interrupted. In addition, transdermal delivery improves and simplifies patient compliance for delivering medication to young children, the elderly and the infirm. The development of these systems poses still scientific and technological challenges. Only an efficient interplay between material scientists, chemists, physicists, and nanotechnologists as well with the medical personal, defining drug doses and pain limits, will push this field further. The move from passive transdermal drug delivery concepts to controllable and on-demand delivery platforms will allow the realization of real personalized point-of-care medication. The clinical potential of light-based transdermal activation using lasers, photodiodes and in combination with light-to-heat converting materials is large. Despite several promising technologies such as the P.L.E.A.S.E. device, the movement of active heat-based transdermal technologies into the market has been slow and not even a hand full of devices has reached the market. The MNs are currently one of the most fashionable drug delivery systems with the main focus on single-dose administration of vaccines. On-demand delivery of drugs *via* light activation might be of high priority in the long run as it will allow treatment of people with chronic diseases such as diabetics, hypertension, etc. The painless delivery of insulin with a feedback control provided by varying glucose levels is something patients as well as the pharmaceutical industry is actively looking for. Photothermal MN technologies as well as photothermal approaches using rGO-based skin patches when combined with blood glucose sampling targeting for a feedback control of drug delivery are the transdermal devices of the future. Having said so, currently part of the research pipeline of several pharmaceutical companies, due to ease of application, increased patient compliance, and safety of transdermal drug delivery systems, is the development of transdermal systems for the delivery of central nervous system and pain-relief medicines. The on-demand aspect is, however, still not integrated into these designed strategies. Considering the progress made in the transdermal field, the future of transdermal drug delivery depends largely on the implementation of novel approaches to overcome constraints of passive diffusion without compromising skin integrity. None of the new nanostructures and thermal approaches are currently FDA approved, a timely process. It is evident that the transdermal market will largely increase due to patient demand for transdermal dosage forms. This will be in conjunction with the adoption of technological approaches that enable the transdermal delivery of a diverse portfolio of therapeutic compounds.

## Author Contributions

SS wrote the second part of the chapter. RB wrote the first part of the chapter.

## Conflict of Interest Statement

The authors declare that the research was conducted in the absence of any commercial or financial relationships that could be construed as a potential conflict of interest.
